# Radioactivity of Tobacco Leaves and Radiation Dose Induced from Smoking

**DOI:** 10.3390/ijerph6020558

**Published:** 2009-02-05

**Authors:** Constantin Papastefanou

**Affiliations:** Aristotle University of Thessaloniki, Atomic and Nuclear Physics Laboratory, Thessaloniki 54124, Greece; E-mail: papastefanou@physics.auth.gr

**Keywords:** Radioactivity, tobacco leaves, radiation dose, smoking

## Abstract

The radioactivity in tobacco leaves collected from 15 different regions of Greece and before cigarette production was studied in order to find out any association between the root uptake of radionuclides from soil ground by the tobacco plants and the effective dose induced to smokers from cigarette tobacco due to the naturally occurring primordial radionuclides, such as ^226^Ra and ^210^Pb of the uranium series and ^228^Ra of the thorium series and/or man-made radionuclides, such as ^137^Cs of Chernobyl origin. Gamma-ray spectrometry was applied using Ge planar and coaxial type detectors of high resolution and high efficiency. It was concluded that the activities of the radioisotopes of radium, ^226^Ra and ^228^Ra in the tobacco leaves reflected their origin from the soil by root uptake rather than fertilizers used in the cultivation of tobacco plants. Lead-210 originated from the air and was deposited onto the tobacco leaves and trapped by the trichomes. Potassium-40 in the tobacco leaves was due to root uptake either from soil or from fertilizer. The cesium radioisotopes ^137^Cs and ^134^Cs in tobacco leaves were due to root uptake and not due to deposition onto the leaf foliage as they still remained in soil four years after the Chernobyl reactor accident, but were absent from the atmosphere because of the rain washout (precipitation) and gravitational settling. The annual effective dose due to inhalation for adults (smokers) for ^226^Ra varied from 42.5 to 178.6 μSv/y (average 79.7 μSv/y), while for ^228^Ra from 19.3 to 116.0 μSv/y (average 67.1 μSv/y) and for ^210^Pb from 47.0 to 134.9 μSv/y (average 104.7 μSv/y), that is the same order of magnitude for each radionuclide. The sum of the effective doses of the three radionuclides varied from 151.9 to 401.3 μSv/y (average 251.5 μSv/y). The annual effective dose from ^137^Cs of Chernobyl origin was three orders of magnitude lower as it varied from 70.4 to 410.4 nSv/y (average 199.3 nSv/y).

## 1. Introduction

Naturally occurring primordial radionuclides of the uranium-radium series, such as ^210^Pb and ^210^Po have long been associated with tobacco plants [1]. Tso *et al*. (1966) [2] stated that the principal mechanism of incorporation involves root uptake from soil and phosphate fertilizers. Francis *et al*. (1968) [3] suggested that the deposition of ^210^Pb by rainfall is the principal mechanism of ^210^Pb entry in plants. The properties and distribution of trichomes (hairs) on tobacco leaf surfaces suggest that they are effective collectors of small Aitken (nuclei) particles (<0.1 μm diameter) by means of diffusive deposition due to Brownian motion of the particles [1, 4, 5]. As about 85 % of tobacco trichomes have glandular heads coated with a sticky exudates mixture of organic compounds, trichomes may retain the small atmospheric particles which are deposited on the glandular heads throughout the period of plant growth.

So, ^226^Ra (^238^U-series), ^228^Ra (^232^Th-series) and ^40^K of the naturally occurring primordial radionuclides which are abundant in soil and in most fertilizers (uranium-238 is associated with phosphate fertilizers [6]) follow root uptake, whereas airborne ^210^Pb, a long-lived (T_1/2_=22.3 y) decay product of radon-222, resides on particles <0.3, μm diameter. Papastefanou and Bondietti [7] reported activity median aerodynamic diameters, AMADs for ^210^Pb associated with aerosol particles ranging from 0.28 to 0.49 μm, averaging 0.37 μm, and is captured on tobacco trichomes.

Cesium-137 and -134 of Chernobyl reactor accident origin, followed both routes, that is, during May 1986 when tobacco plants were planted, both radionuclides were in high concentration in air (1.95 and 0.98 Bq m^−3^, respectively [8]) and these radionuclides were deposited onto the plant leaves and captured on tobacco trichomes. In the years which followed, cesium and all fallout radionuclides after dry and even wet deposition onto the ground were mixed and migrated in the soil to deeper layers below the ground surface, suggesting root uptake.

The issue of radioactivity in tobacco smoke has received much attention in the scientific press and, increasingly, in the medical press [9]. Besides this, Martell and Sweder [10] reported that radon decay products naturally found in the environment are altered when they pass through burning cigarettes into mainstream smoke. Martell [11] stated that the cumulative alpha-, beta- and gamma-radiation dose, particularly that from alpha radiation, from inhaled radionuclides deposited in small volumes of bronchial tissue may be an important factor in the initiation of bronchial (lung) cancer in smokers. The smokers are subjected to alphas radiation in the bronchial epithelium from three sources: (1) from indoor radon and thoron decay products inhaled between cigarettes, (2) from ^214^Po, ^212^Po and ^212^Bi in large mainstream smoke particles and (3) from ^210^Po which grows from decay of ^210^Pb-enriched particles that persist at bronchial bifurcations [12].

Radford Jr and Hunt [13] at Harvard School of Public Health (Boston, MA,) reported that for an individual smoking two packages of cigarettes a day, the radiation dose to bronchial epithelium from ^210^Po inhaled in cigarette smoke probably is at least seven times that from background sources, in localized areas may be up to 10 Sv (1000 rem) or more in 25 y. Besides, Winters and Di Franza [14] at the University of Massachusetts (Boston, MA) much later reported that in a person smoking one and a half packs of cigarettes (i.e. 30 cigarettes) per day, the radiation dose to the bronchial epithelium in areas of bifurcation is 80 mSv y^−1^ (8,000 mrem) – the equivalent of the dose to the skin from 300 X-ray films of the chest per year. This figure was comparable with total-body exposure to natural background radiation containing 0.8 mSv y^−1^ (80 mrem) in someone living in the Boston area.

Takizawa *et al*. [15] reported that the range of ^210^Po contained in the tobacco grands in Japan varied from 13.0 to 20.1 Bq kg^−1^ (mean 15.4 Bq kg^−1^), 50 % of ^210^Po present in tobacco was transferred into the smoke and the other 50 % remained in the ash and butt. One pack-a-day smoker inhaled 24 mBq d^−1^ of ^210^Po through smoking and the annual inhalation was 8.8 Bq. Peres and Hiromoto [16] reported that ^210^Po in (dry) tobacco in Brazil ranged from 10.9 to 27.4 Bq kg^−1^ and ^210^Pb from 11.9 to 30.2 Bq kg^−1^. The collective committed effective dose resulting from the use of cigarettes produced in Brazil per year was estimated to be 1.5x10^4^ man Sv, considering an annual production of 5x10^8^ kg of cigarettes in Brazil and the committed effective dose of 0.16 mSv y^−1^ of cigarette smoking.

Colangelo *et al*. [17] reported ^210^Po in tobacco in Argentina ranged from 10 to 80 Bq kg^−1^ and the lung dose due to the use of tobacco varied from 75 to 600 μSv y^−1^. Khater [18] reported that the range of ^210^Po in cigarette tobacco in Egypt ranged from 9.7 to 22.5 mBq per cigarette (average 16.6 mBq per cigarette). The average percentages of ^210^Po content in fresh tobacco plus wrapping paper that were recorded by post-smoking filters, ash and smoke were 4.6, 20.7 and 74.7, respectively. Cigarette smokers, who are smoking one pack (20 cigarettes) per day, are inhaling on average 123 mBq d^−1^ of ^210^Po and ^210^Pb each. The mean values of the annual effective dose for smokers (one pack per day) were estimated to be 193 and 251 μSv y^−1^ from ^210^Po and ^210^Pb, respectively.

Approximately 10 L d^−1^ is inhaled through 40 cigarettes-this is about 1/2000 of the amount of air usually breathed per day (20 m^3^ d^−1^ [10, 19]. Published radiochemical data for inhaled mainstream smoke showed on average ^210^Po content of 1.33 mBq (0.036 pCi) per cigarette or 96.2 Bq kg^−1^ smoke (2.6 pCi ^210^Po per g smoke) [20], with a ^210^Pb: ^210^Po ratio of 0.66 ± 0.23 [21].

This paper reports data on the radioactivity of tobacco leaves after the collection from tobacco fields before cigarette production in order to find any association in the uptake of the naturally occurring radionuclides and of cesium radioisotopes of Chernobyl origin, and to estimate the effective dose from cigarette tobacco as the cumulative α-, β- and γ-radiation dose, particularly that from α-radiation, from inhaled radionuclides deposited in small volumes of bronchial tissue may be an important factor in the initiation of bronchial (lung) cancer in smokers [11].

## 2. Materials and Methods

**Figure 1.**
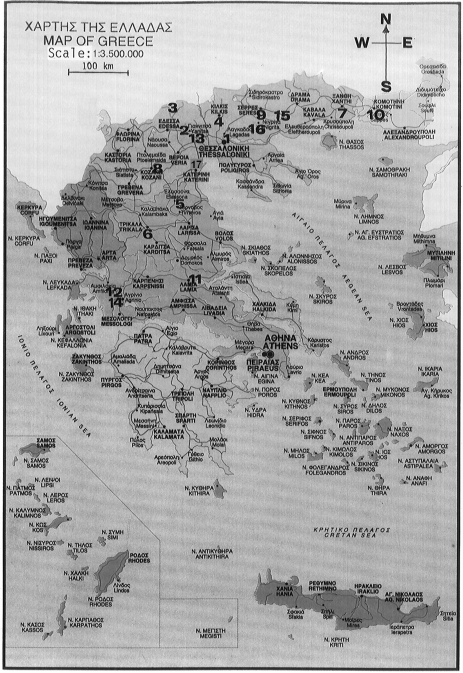
A map of Greece showing the sampling locations of the tobacco leaves (Table 1).

Seventeen different samples of tobacco leaves produced in the year 1990 at different locations in Greece (Figure 1, Table 1) were examined a year later for radioactivity using γ-ray spectrometry. The spectrometric system consisted of two different high-purity Germanium low-background detectors. One planar Ge detector of active area 2000 mm^2^, thickness 20 mm, Be window 0.6 mm and energy resolution (FWHM) 400 eV for 5.9 keV γ-rays (^55^Fe) and 700 eV for 122 keV γ- rays (^57^Co), was appropriate for γ-rays ranging from 5 to 186 keV for determination of ^210^Pb (47 keV), ^226^Ra (186 keV) and ^238^U via its decay product ^234^Th with 63 and 93 keV γ-rays. A second coaxial Ge detector (p-type), crystal size 155 cc, resolution (FWHM) 1.9 keV at 1.33 MeV (^60^Co) and 900 eV at 122 keV (^57^Co), peak-to-Compton ratio 55:1 and efficiency 42 %, was appropriate for γ-rays ranging from 240 to 2614 keV for determination of ^226^Ra via its decay products ^214^Pb and ^214^Bi, ^232^Th via its decay products ^228^Th, ^228^Ac and ^228^Ra, ^40^K (1460 keV), ^137^Cs (662 keV) and ^134^Cs (605 keV).

The samples were dried before radioactivity measurement for 3–4 days at a temperature of 30°C to avoid any moisture adsorption. After that, the samples were cut into very small pieces using a blender and were mixed with active charcoal and then sealed appropriately for about one month to reduce leaching and to attain radioactive equilibrium between radon and thoron decay products [22], as eight half-lives of ^222^Rn (T_1/2_=3.82 d), the decay product of ^226^Ra and precursor of ^214^Pb and ^214^Bi, correspond to 1 month. About eight half-lives of ^224^Ra (T_1/2_=3.66 d), the decay product of ^228^Ra, are also 1 month.

The samples were measured for radioactivity in two geometries, that is, in a standard geometry 40 g plastic can of 6 cm diameter and a Marinelli beaker of 1 L (volume). The overall efficiency of the counting system was known to an accuracy of better than 5 % for the plastic can geometry and about 12 % for the Marinelli beaker. The +− errors in Table 1 are the experimental errors of each measurement. The collecting time was 120,000 s.

**Figure 2.**
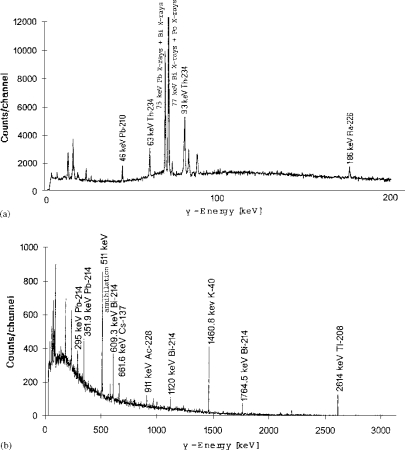
Gamma-ray spectrum of tobacco leaves. (a) Gamma photon energies ranging up to 186 keV obtained with planar detector. (b) Gamma photon energies ranging up to 2614 keV obtained with coaxial detector.

Gamma-ray spectra obtained with both HPGe detectors are presented in Figure 2a for the planar detector and Figure 2b for the coaxial detector.

## 3. Results and Discussion

### 3.1. Radionuclide Concentrations

Table 1 presents the concentrations in Bq kg^−1^ of ^226^Ra, ^210^Pb, ^228^Ra, ^40^K, ^137^Cs and ^134^Cs in Greek tobacco leaves produced in 1990, i.e. four years after the Chernobyl reactor accident (26 April 1986).

In the tobacco leaves, ^226^Ra activity concentrations ranged from 1.80 to 7.57 Bq kg^−1^ (average 3.38 Bq kg^−1^), while ^228^Ra activity concentrations ranged from 1.10 to 6.52 Bq kg^−1^ (average 3.83 Bq kg^−1^). The results showed that ^226^Ra and ^228^Ra concentrations in tobacco leaves were comparable, reflecting their origin in soil by root uptake rather than in fertilizers used for cultivation in the fields. It is known that ^226^Ra in soil ranges from 17 to 60 Bq kg^−1^ (average 35 Bq kg^−1^), ^238^U in soils ranges from 16 to 110 Bq kg^−1^ (average 35 Bq kg^−1^) and ^228^Ra (^232^Th) in soils ranges from 11 to 64 Bq kg^−1^ (average 30 Bq kg^−1^) [23]. Papastefanou (2001) [6] reported that ^238^U concentrations in phosphate fertilizers ranged from 312 to 936 Bq kg^−1^ (average 638 Bq kg^−1^). Radium-226 concentrations in phosphate fertilizers ranged from 19 to 1129 Bq kg^−1^ (average 418 Bq kg^−1^). Thorium-232 concentrations in phosphate fertilizers ranged from 2 to 12 Bq kg^−1^ (average 6 Bq kg^−1^).

Lead-210 activity concentrations in the tobacco leaves ranged from 6.34 to 18.2 Bq kg^−1^ (average 14.12 Bq kg^−1^). The results showed that ^210^Pb concentrations were much higher than those of ^226^Ra, indicating that ^210^Pb atoms originating from the air were deposited onto the leaves of the tobacco plants and were trapped by the trichomes. It must be noted that ^210^Po, an α-emitter (T_1/2_=138.38 d) and decay product of ^210^Pb, is a constituent of ambient aerosol particles and must be deposited onto the tobacco leaves and trapped by the trichomes also.

Potassium-40 activity concentrations in the tobacco leaves ranged from 273 to 2080 Bq kg^−1^ (average 823 Bq kg^−1^). Two of the seventeen samples (Table 1) showed unusually high activity concentrations of ^40^K. The results showed that ^40^K concentrations were due to root uptake either from soil or from fertilizer. Potassium-40 in soils ranges from 140 to 850 Bq kg^−1^ (average 400 Bq kg^−1^) [23] and in phosphate fertilizers from 53 to 6370 Bq kg^−1^ (average 2438 Bq kg^−1^) [6].

Cesium-137 activity concentrations in the tobacco leaves ranged from 1.20 to 7.00 Bq kg^−1^ (average 3.40 Bq kg^−1^). The results showed that ^137^Cs concentrations were due to root uptake because ^137^Cs (T_1/2_=30.17 y) still remained in soil four years after the Chernobyl reactor accident [24]. Cesium-137 concentrations in tobacco leaves in 1986, the year of Chernobyl accident, varied between 70 and 180 Bq kg^−1^ (average 100 Bq kg^−1^). According to fallout radioactivity measurements performed by the Atomic and Nuclear Physics Laboratory, Aristotle University of Thessaloniki for the Ioannou Tobacco Industry, at Thessaloniki, Northern Greece, as reported on April 12, 1987 on a special certificate used for dry tobacco export.

### 3.2. Effective Dose Estimate

Assuming 0.82 g of tobacco per cigarette in Greek cigarettes and a smoker is smoking 30 cigarettes (one and a half packs) per day or 24.6 g of tobacco per day, then the annual consumption of tobacco by cigarettes is estimated to be 8.985 kg y^−1^. Taking into consideration the data of Table 1 for the radionuclide concentrations (Bq kg^−1^) in tobacco dry leaves, the fraction of the radionuclide activity concentration that is recovered from cigarette tobacco to cigarette smoke is 0.75 (75 %), as on the average, about 75 % of the radioisotope in the cigarette tobacco was contained in the cigarette smoke, which is partially inhaled and deposited in the lung tissues and about 25 % was retained in the cigarette filter and ash [18] and the most recent dose conversion coefficients of the radionuclides (Sb Bq^−1)^ for the case of inhalation for adults (smokers) as presented in Table 2, then the data of Table 3 are derived for the annual effective dose, H_E_ (Sv y^−1^), due to inhalation for adults (smokers), according to the equation:

(1) $${\text{H}}_{\text{E}}=0.75\;\text{x}\;{\text{M}}_{\text{T}}\;\text{x}\;\text{Ci}\;\text{x}\;\text{F}$$

where M_T_ (kg y^−1^) refers to the annual amount (in mass) of tobacco consumed, Ci (Bq kg^−1^) refers to the concentration of the ith radionuclide, and F (Sv Bq^−1^) refers to the dose conversion factor [25–28].

From the data of Table 3, it is shown that the annual effective dose to ^226^Ra varied from 42.5 to 178.6 μSv^−1^ (average 79.7 μSv y^−1^), while for ^228^Ra from 19.3 to 116.0 μSv y^−1^ (average 67.1 μSv y^−1^) and for ^210^Pb from 47.0 to 134.9 μSv y^−1^ (average 104.7 μSv y^−1^), that is of the same order of magnitude for each natural radionuclide. The dose from ^210^Po (α-emitter), a decay product of ^210^Pb, should be of the same order of magnitude of the dose due to ^210^Pb. Holtzman and Ilcewicz (1966) [21] reported that in smokers the ^210^Po is nearly in radioactive equilibrium with its precursor ^210^Pb (^210^Po:^210^Pb=0.87±0.10). The sum of the effective doses of the three natural radionuclides varied from 151.9 to 401.3 μSv y^−1^ (average 251.5 μSv y^−1^). This dose must be compared with the average worldwide exposure to natural radiation sources 2.4 mSv y^−1^ and especially the part due to inhalation which is 1.26 mSv y^−1^ [23].

The annual effective dose due to ^137^Cs of Chernobyl origin was three orders of magnitude lower. It varied from 70.4 to 410.4 nSv y^−1^ (average 199.3 nSv y^−1^) and so very little is contributing to the total dose due to inhalation to smokers. Cesium-134, with a relatively short half-life (T_1/2_=2.6 y) was not considered in estimating the annual effective dose for its faster decay resulting in activity concentrations mostly below the maximum detectable activity, MDA (Table 1).

Dose induced from ^40^K was not considered, although its concentration in all samples of tobacco leaves was of the same level of this radionuclide in the soil ground except that of the samples No. 13 and 14 (Table 1). Potassium is one of the most abundant element in every environmental sample. Potassium-40 is the predominant radioactive component in normal food and human tissue and distributes throughout the human body [19].

From the literature, it is seen that the dose estimated for smokers was considered only for ^210^Po (α-emitter) and its precursor ^210^Pb (β-, γ-emitter). No consideration was taken on the radioisotopes of radium, i.e. ^226^Ra of the uranium series and ^228^Ra of the thorium series, although the dose for inhalation from each of them was shown to be of the same order of magnitude with that of ^210^Pb and of course, of ^210^Po [27–28].

For the dose due to ^137^Cs of Chernobyl origin, Fletcher (1994) [29] was referred and concluded that the annual effective dose for inhalation of ^137^Cs was estimated to be 7.44 nSv y^−1^ with the ^137^Cs content in tobacco leaves averaging to 40 Bq kg^−1^, in Libya and annual intake 1 Bq y^−1^ for ^137^Cs.

## 4. Conclusions

The radioactivity in tobacco leaves collected from 15 different regions of Greece before cigarette production has been studied in order to find any association between the uptake of the naturally occurring primordial radionuclides and the radioisotopes of cesium of Chernobyl origin. The activities of the radioisotopes of radium, ^226^Ra and ^228^Ra in the tobacco leaves would be either through root uptake or through the fertilizers used for cultivation of tobacco plants in the fields, as the air concentrations of the radioisotopes of radium are extremely low. Lead-210 originating from air was deposited onto the tobacco leaves and trapped by the trichomes. Potassium-40 in the tobacco leaves was due to root uptake either from soils or from fertilizers. The radioisotopes of cesium, ^137^Cs and ^134^Cs, in the tobacco leaves were due to root uptake and not to deposition onto the leaf foliage as they still remained in soil four years after the Chernobyl reactor accident but were absent from the atmosphere because of the rain washout (precipitation) and gravitational settling.

In estimating the radiation dose induced from smoking, it was concluded that the annual effective dose to lungs due to inhalation for adults (smokers) averaged to 80 μSv for ^226^Ra, 67 μSv for ^228^Ra and 105 μSv for ^210^Pb, that is 252 μSv in total. The annual effective dose due to ^137^Cs of Chernobyl origin averaged to about 200 nSv, that is three orders of magnitude lower than that of the naturally occurring radionuclides. The effective dose of 252 μSv per year must be compared with the average worldwide exposure to natural radiation sources due to inhalation 1.26 mSv y^−1^.

## Figures and Tables

**Table 1.** Activity concentrations in tobacco leaves (Bq kg-1).

No	Lab.No.	^226^Ra	^210^Pb	^228^Ra	^40^K	^137^Cs	^134^Cs
1	TAB-2	6±1	16±1	5±1	819±20	7±1	M.D.A
2	TAB-3	2±1	16±5	6±1	897±21	4±1	M.D.A
3	TAB-4	3±2	17±5	5±1	619±270	6±1	M.D.A.
4	TAB-5	4±1	18±4	5±1	825±20	7±1	M.D.A.
5	TAB-6	3±1	9±4	5±1	771±19	5±1	M.D.A.
6	TAB-7	2±1	6±4	3±1	872±16	6±1	M.D.A.
7	TAB-8	8±1	14±5	7±1	967±22	2±1	M.D.A.
8	TAB-9	3±1	16±5	3±1	618±16	3±1	-
9	TAB-10	3±1	18±5	5±1	745±19	2±1	M.D.A.
10	TAB-11	3±1	14±4	4±1	751±14	3±1	M.D.A.
11	TAB-12	2±1	17±5	1±1	756±20	2±1	M.D.A
12	TAB-13	4±1	8±5	3±1	879±18	2±1	M.D.A.
13	TAB-14	3±1	16±5	3±1	2080±26	2±1	M.D.A.
14	TAB-15	2±2	17±4	3±1	1110±20	M.D.A.	M.D.A.
15	TAB-16	3±1	15±5	4±1	493±16	1±1	M.D.A
16	TAB-17	3±1	7±4	2±1	273±13	2±1	M.D.A
17	TAB-18	3±1	17±4	4±1	503±16	4±1	0.6±0.2

**Table 2.** Dose conversion factors for inhalation for adults.

Radionuclide	Sv Bq^−1^	Reference
^226^Ra	3.50x10^−6^	[27–28]
^228^Ra	2.60x10^−6^	[27–28]
^210^Pb	1.10x10^−6^	[27–28]
^210^Po	3.30x10^−6^	[27–28]
^137^Cs	8.70x10^−9^	[25–26]

**Table 3.** Annual effective dose of smokers in smoking 30 cigarettes per day.

No.	Lab.No.	^226^Ra (μSv y^−1^)	^210^Pb (μSv y^−1^)	^228^Ra (μSv y^−1^)	^137^Cs (nSv y^−1^)
1	TAB-2	146.47	121.57	89.36	410.39
2	TAB-3	50.24	114.89	98.47	235.68
3	TAB-4	74.06	122.31	83.93	367.01
4	TAB-5	86.33	134.91	88.83	410.39
5	TAB-6	70.28	63.90	84.28	275.55
6	TAB-7	55.67	47.00	53.61	371.69
7	TAB-8	178.55	106.74	115.99	99.67
8	TAB-9	76.89	119.34	55.01	175.88
9	TAB-10	74.77	129.72	81.12	93.80
10	TAB-11	65.89	104.52	72.19	191.12
11	TAB-12	42.46	127.50	19.28	99.67
12	TAB-13	86.80	56.48	52.74	123.12
13	TAB-14	73.59	119.34	46.96	124.29
14	TAB-15	51.42	128.98	51.34	—
15	TAB-16	77.36	111.93	61.67	70.35
16	TAB-17	73.12	47.96	30.84	89.70
17	TAB-18	74.06	122.31	61.15	249.17
